# Supplementary material for: Targeted Delivery of BZLF1 to DEC205 Drives EBV-Protective Immunity in a Spontaneous Model of EBV-Driven Lymphoproliferative Disease
Source: Vaccines (Basel). 2021 May 26;9(6):555. doi: 10.3390/vaccines9060555 (PMC8228306; doi:10.3390/vaccines9060555)
Supplement: Supplementary file 1 [file vaccines-09-00555-s001.zip › vaccines-1208208-supplementary.pdf]

Targeted Delivery of BZLF1 to DEC205 Drives EBV-Protective Immunity in a Spontaneous Model of EBV-Driven Lymphoproliferative Disease

Elshafa H. Ahmed[1, 2], Eric Brooks[2], Shelby Sloan[1, 2], Sarah Schlotter[2], Frankie Jeney[2], Claire Hale[3], Charlene Mao[2], Xiaoli Zhang[4], Eric McLaughlin[4], Polina Shindiapina[2, 5], Salma Shire[6], Manjusri Das[2], Alexander Prouty[2], Gerard Lozanski[7], Admasu T. Mamuye[8], Tamrat Abebe[9], Lapo Alinari[2,5], Michael A. Caligiuri[10], and Robert A. Baiocchi[2, 5]

Figure S1

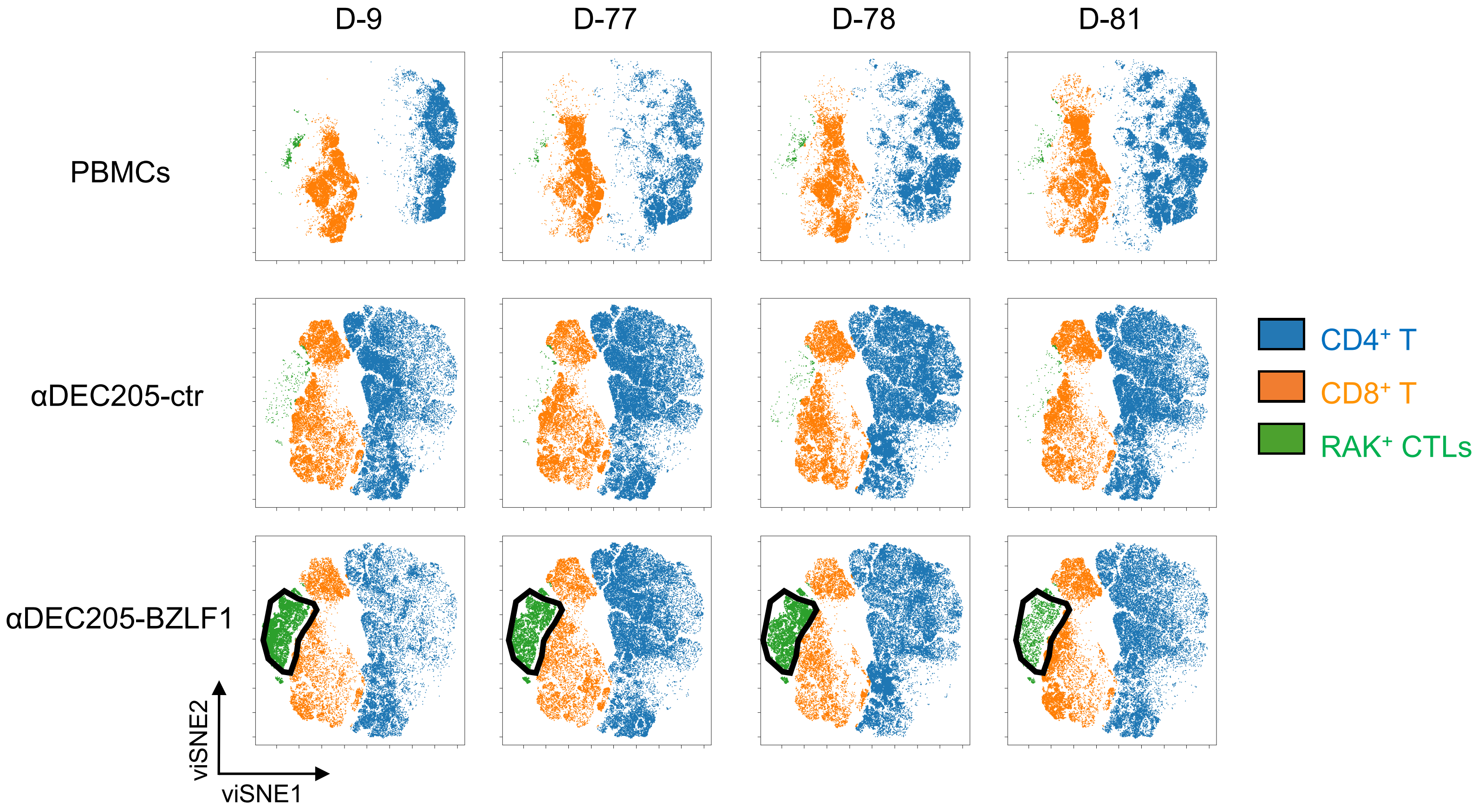

Figure S1. viSNE immunome maps of $CD3^+$ T-cells showed remarkable expansion within the viSNE space populated of the $CD3^+CD8^+RAK^+$ from DEC205-BZLF1 co-culture across 4 HLA-B8 donors.

Figure S2

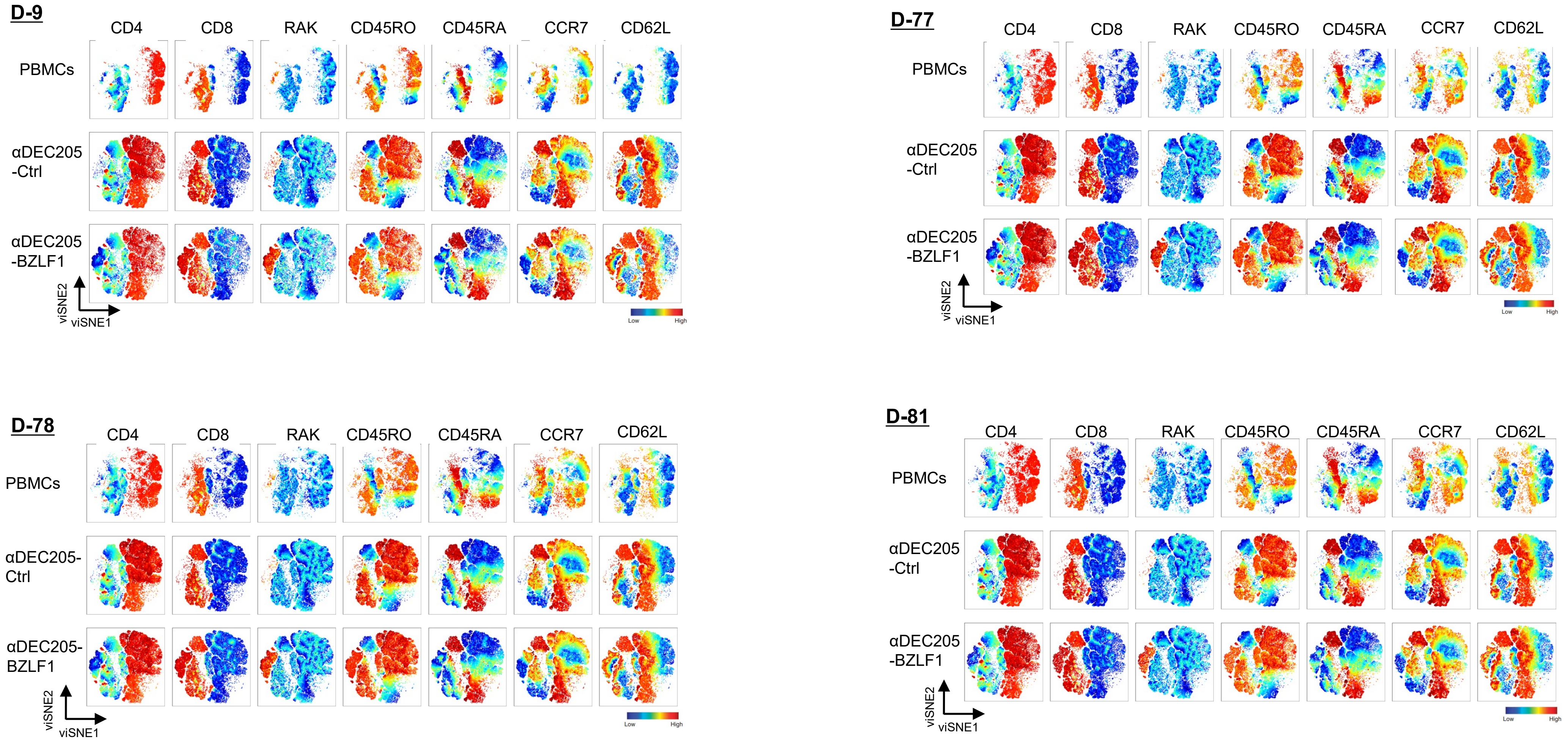

Figure S2. The viSNE maps, colored by the intensities of individual immune marker expression (CD4, CD8, RAK, CD45RO, CD45RA, CCR7 and CD62) for 4 HLA-B8 donors.

Figure S3

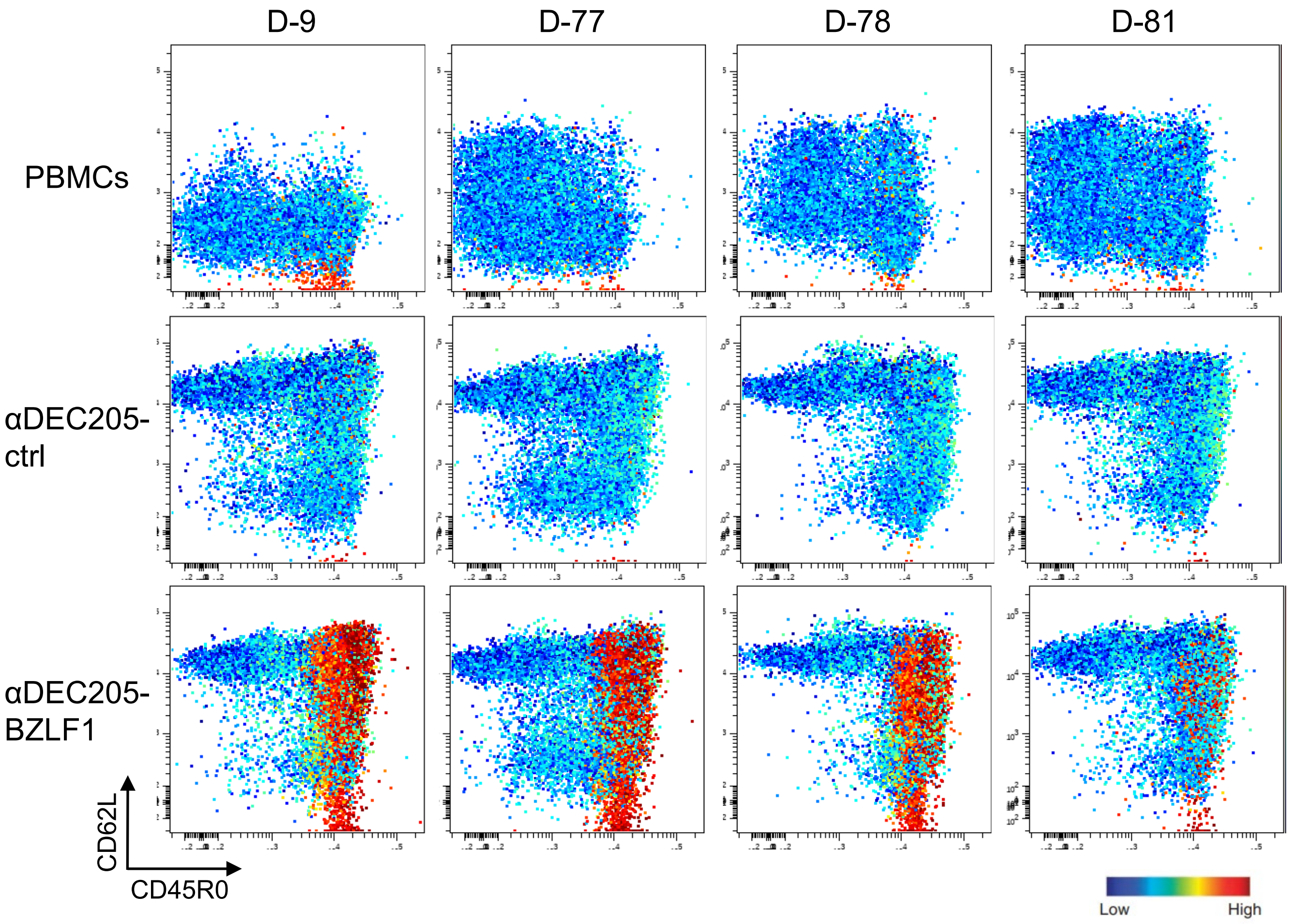

Figure S3. Biaxial plots colored according to the intensity of the RAK marker from 4 HLA-B8 donors.

Figure S4

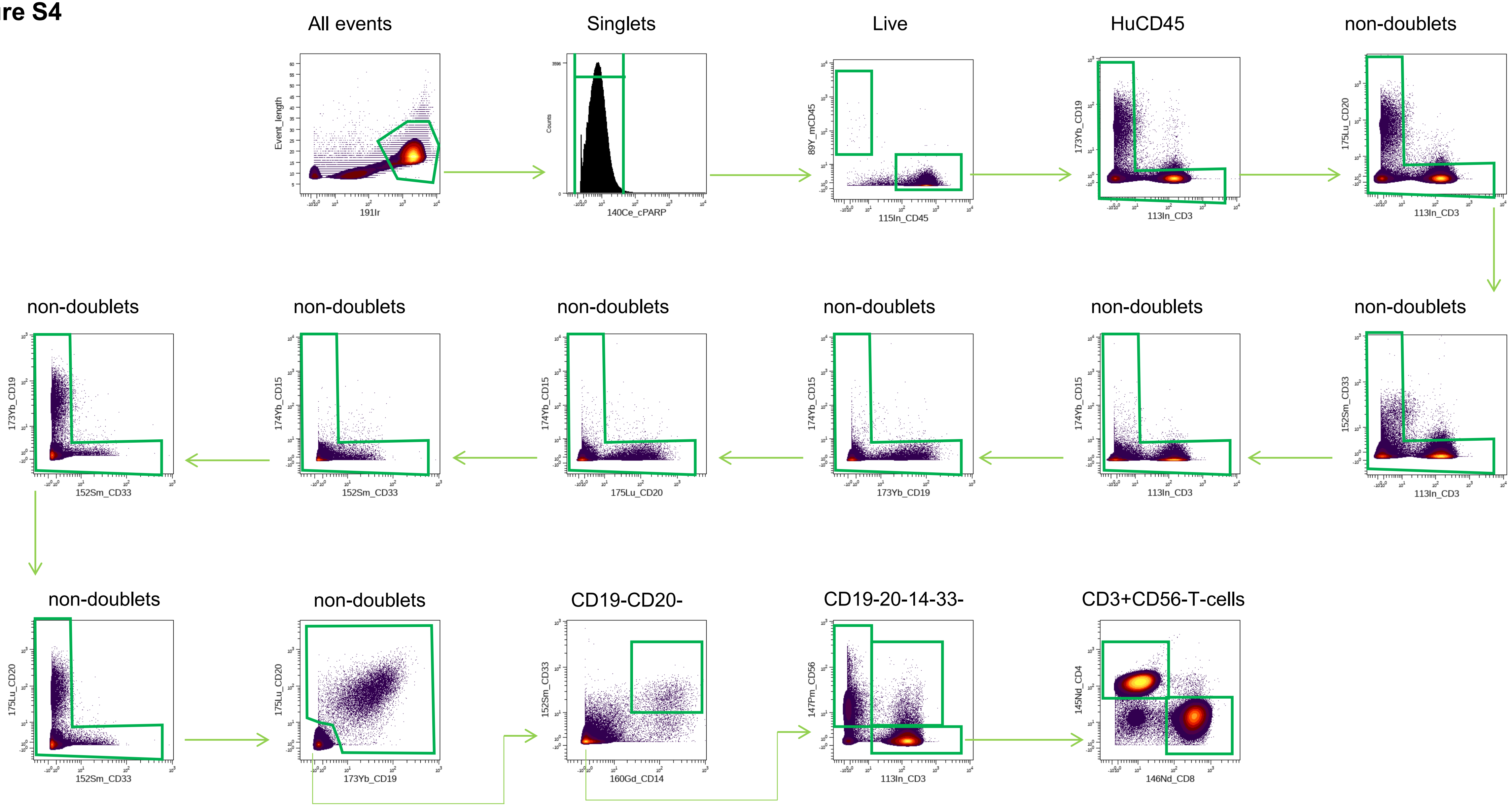

Figure S4. Gating Hierarchy for cell events on samples analyzed by mass cytometry (health donor PBMCs, cells produces on the co-culture and splenocytes from SCID mice vaccinated with αDEC205-BZLF1 and αDEC205-Ctrl). Cell events for normal sample #D9 are shown. For *in vivo* samples, murine CD45 population was excluded first. Then non-doublets cells were sub-gates and the lineage populations were defined as shown.

Figure S5

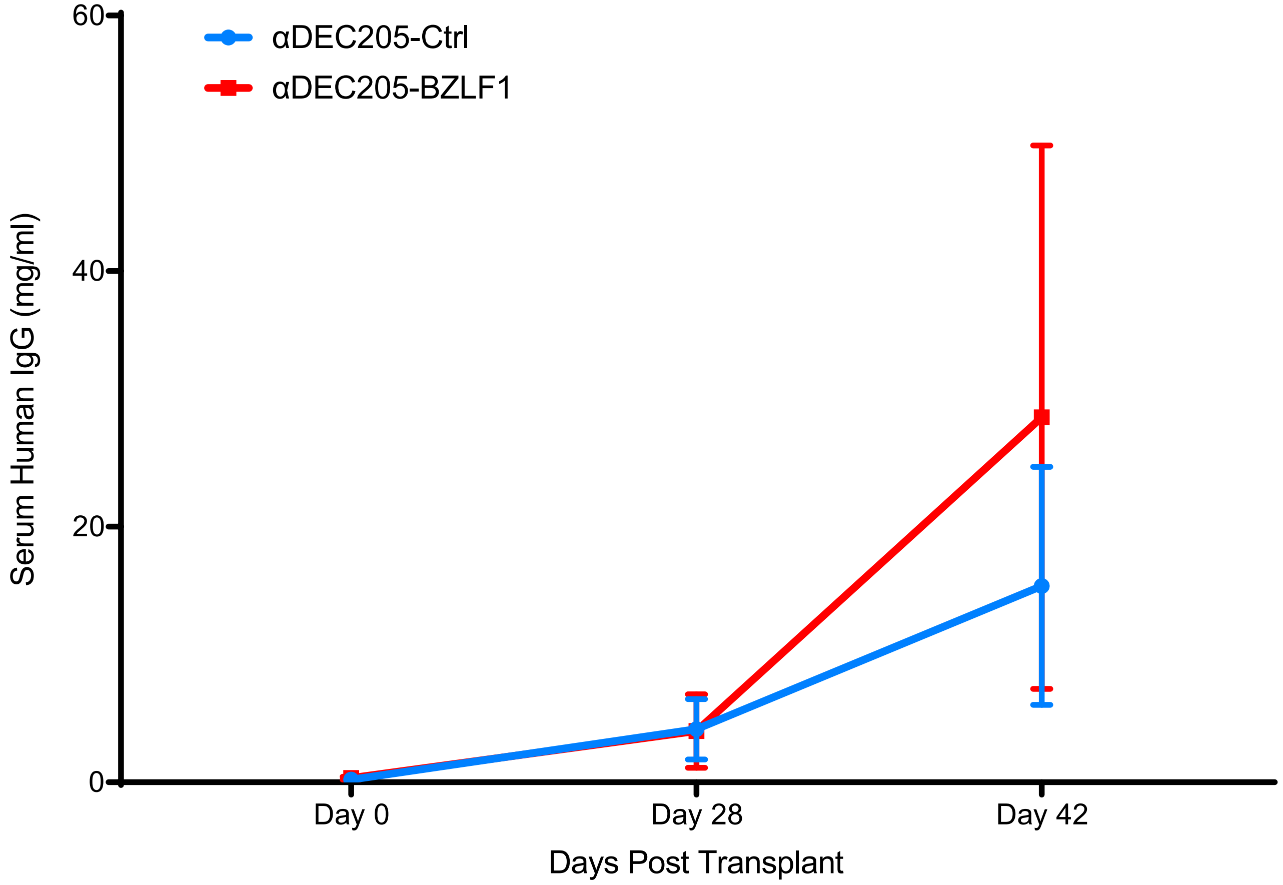

Figure S5. ELISA assay measuring human IgG in mouse serum from αDEC205-BZLF1 and αDEC205-Ctrl vaccine groups.

Table S1. List of antibodies for CyTOF analysis

Ab	Metal	Clone	Company
mCD45	Y89	30F11	Fluidigm
CD3	113In	HIR2	Biolegend
CD45	115In	HI30	Biolegend
IFNγ	139La	B27	Biolegend
cPARP	140Ce	F21-852	BD Pharmingen
CCR6	141Pr	G034E3	Biolegend
CD107a	142Nd	H4A3	Biolegend
T-bet	143Nd	eBio4B10	eBioscience
CD94	144Nd	HP-3D9	BD Biosciences
CD4	145Nd	RPA-T4	Biolegend
CD8	146Nd	RPA-T8	Biolegend
CD56	147Sm	NCAM16.2	BD Biosciences
CD154 (CD40L)	148Nd	24-31	Biolegend
CD127	149Sm	A019D5	Biolegend
CCR4	150Nd	205410	R&D systems
ICOS	151EU	DX29	BD Biosciences
CD33	152Sm	P67.6	Biolegend
HLA-DR	153Eu	L243	Biolegend
CD69	154Sm	FN50	Biolegend
PD-1	155Gd	EH12.2H7	Biolegend
CXCR3	156Gd	G025H7	Fluidigm
CD62L	157Gd	DREG-56	Biolegend
CD27	158Gd	L128	Fluidigm
CXCR5	159Tb	J252D4	Biolegend
CD14	160Gd	M5E2	Biolegend
CD16	161Dy	3G8	Biolegend
Foxp3	162Dy	PCH101	Fluidigm
GATA3	163Dy	16E14A23	Biolegend
GITR	164Dy	108-17	Biolegend
CD45RO	165Ho	UCHL1	Biolegend
NKG2D	166Er	1D11	Biolegend
CCR7	167Er	G043H7	Fluidigm
RORgt	168Er	600214	Fluidigm
CD25	169Tm	2A3	BD Biosciences
CTLA-4	170Er	14D3	eBioscience
GRZB	171Yb	QA16A02	Biolegend
PD-L1	172Yb	24F.10C12	Fluidigm
CD19	173Yb	H1B19	BD Biosciences
CD15	174Yb	W6D3	Biolegend
CD20	175Lu	2H7	Biolegend
Bcl6	176Yb	IG191E/A8	Biolegend
CD11b	209Bi	ICRF44	Biolegend
